# Genomic characterisation of endangered Landim pigs reveals distinctive features and immune-related selection

**DOI:** 10.3389/fvets.2025.1633365

**Published:** 2025-10-14

**Authors:** Fábio Teixeira, Pedro Sá, Dulce Santos, Carmen Garrine, Rosa Zimba, Laurinda Augusto, Endika Varela-Martínez, Hermenegildo Chiaia, Kiala Sebastino, José M. Cordeiro, Alexandre Leitão, Luís T. Gama, Andreia J. Amaral

**Affiliations:** ^1^Universidade de Évora, MED (Mediterranean Institute for Agriculture, Environment and Development) & CHANGE (Global Change and Sustainability Institute), Évora, Portugal; ^2^Faculty of Veterinary Medicine, University José Eduardo dos Santos, Huambo, Angola; ^3^Animal Breeding and Genomics, Wageningen University and Research, Wageningen, Netherlands; ^4^BE, Bioinsight & Ecoa, Odivelas, Portugal; ^5^Faculty of Veterinary, Eduardo Mondlane University, Maputo, Mozambique; ^6^Faculty of Veterinary Medicine, Lusófona University, Lisbon, Portugal; ^7^Escola Superior de Desenvolvimento Rural de Vilankulo, University Eduardo Mondlane, Inhambane, Mozambique; ^8^Department of Genetics, Physical Anthropology and Animal Physiology, Faculty of Science and Technology, University of the Basque Country (UPV/EHU), Leioa, Spain; ^9^CIISA - Centre for Interdisciplinary Research in Animal Health, Faculty of Veterinary Medicine, University of Lisbon, Lisbon, Portugal; ^10^AL4AnimalS - Associate Laboratory for Animal and Veterinary Sciences, Lisbon, Portugal; ^11^IIV - Instituto de Investigação Veterinária, Huambo, Angola; ^12^Department of Zootechny, School of Science and Technology, University of Evora, Évora, Portugal

**Keywords:** pigs, selection, Landim, whole-genome sequencing, innate immunity

## Abstract

**Introduction:**

The Landim pigs of Mozambique are an important local breed, reared by smallscale farmers in extensive systems, predominantly sustained on leftover food. These pigs serve as a cornerstone of the economic stability of these communities. However, their existence is currently threatened by uncontrolled crossbreeding with commercial pig breeds. Preserving these local pigs is also crucial, given their role as a source of genetic diversity.

**Methods:**

Whole-genome resequencing of samples derived from six Landim pigs,was conducted, to characterise their genetic makeup and establishing their relationship with pig breeds worldwide.

**Results:**

Our findings suggest that Landim pigs are more closely related to Angolan pigs, although recent introgression from European cosmopolitan breeds has occurred.

**Discussion:**

Results show that Landim pigs display a unique genetic signature, with positive selection detected in variants of genes acting within the neuroimmune axis, which is fundamental for survival and immune response.

## 1 Introduction

**Figure 1:**
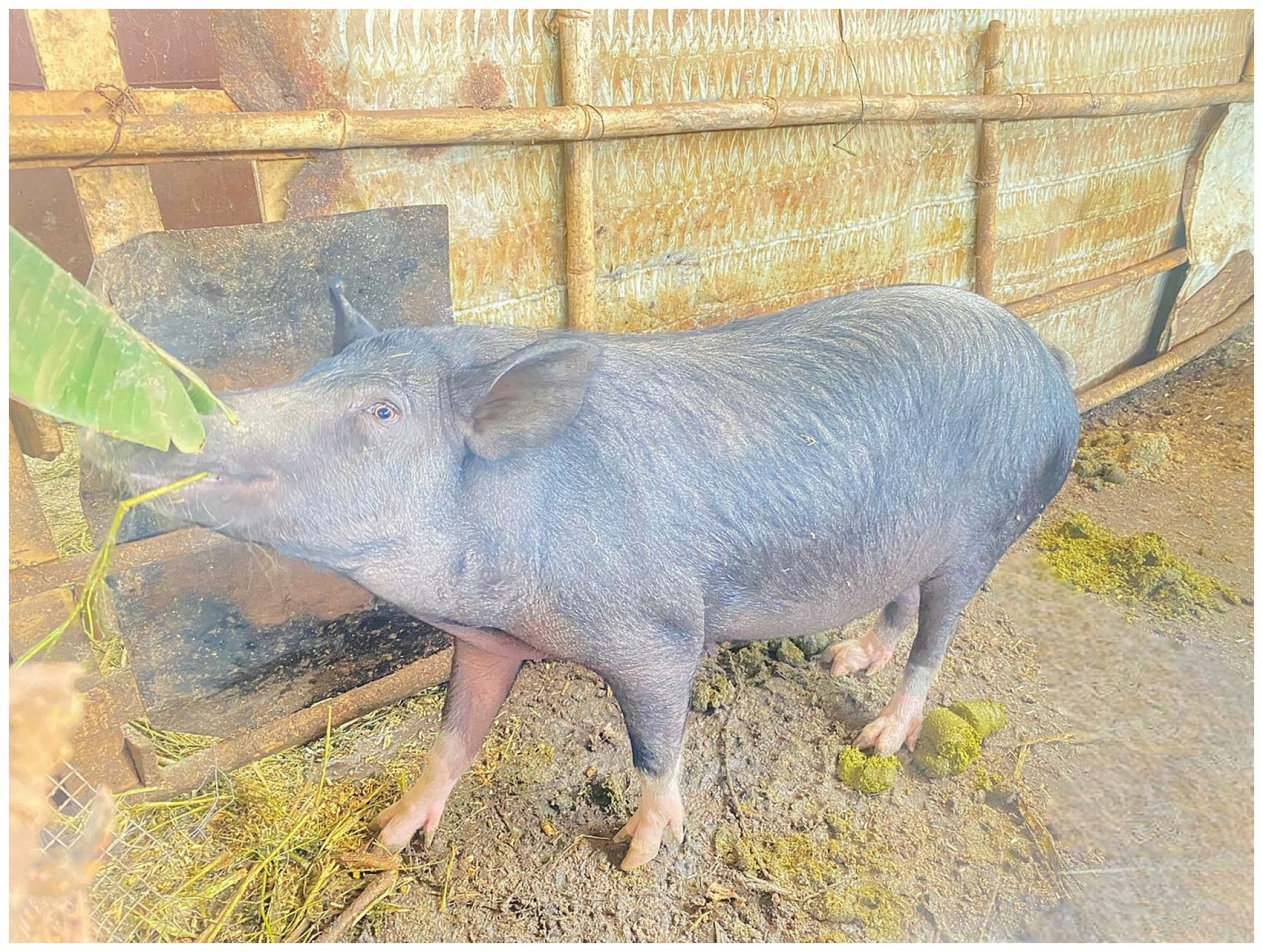
Landim pig from Mozambique.

Pigs are a very important protein source for humans, and in 2021, they represented 33% of the total meat production worldwide (1). In many developing countries, pigs are reared as an essential income source, as in sub-Saharan African countries, where smallholders keep most pigs (2). Local breeds, such as the Landim pigs of Mozambique, are usually kept in extensive production systems, where they are fed by-products from crops that cannot be consumed or used more efficiently by households (3). Small producers, primarily women, rear these pigs free-ranged and confined at night or during the rainy season. They are mainly fed maize bran, watermelons, cabbage, and sweet potato leaves and have no access to healthcare (4, 5). Landim pigs also have the capacity to survive disease outbreaks (6). In 2020, pig production represented nearly 40% of all meat production in Mozambique, with 92 thousand tonnes produced (7). The colour phenotype of Landim pigs is quite diverse; animals can be black, brown, or brown with spots. In relation to other morphological characteristics, Landim pigs have relatively long legs, small erect ears, an elongated snout, and a short curled or straight tail (8) (Figure 1). These pigs have an average litter size of 6 to 10 piglets (R. Zimba, personal communication, December 2024) and an average daily gain of 120 g from 0 to 8 months (9).

In Mozambique, the pig population is sometimes affected by African swine fever (ASF), which may eliminate most animals. However, records exist that Landim pigs might be resistant to ASF (6). Landim pigs are known as a locally adapted breed, but they are experiencing a reduction in census due to crossbreeding with exotic commercial lines. The existing population is kept in small herds, which are vulnerable to inbreeding and natural disasters. Therefore, there is a need to address research and policy gaps to formulate strategies for the conservation of local breeds (10). The genetic characterisation of native genetic resources is a preliminary step for developing conservation programmes that will enable the promotion of local breeds and support their sustainable use (11). Moreover, the possible identification of critical genetic variants related to productive and/or adaptive traits can provide valuable information for farmers, enabling them to make informed breeding decisions and preserve the genetic diversity of local breeds (12).

In recent years, the possibility of generating whole-genome sequencing (WGS) data cost-effectively has enabled the availability of WGS for many breeds around the world (13–15), providing novel insights into the relationships between different world breeds and the effects of selection. These studies, however, have mostly focused on European and Asian pig breeds. More recently, some studies have started to contribute to the study of African pig breeds, and WGS data are currently available for local pigs from Angola (16), Kenya and Nigeria (17). The goal of this study was to understand the origins of native pigs from Mozambique, based on a sample of individuals collected in the Inhambane province. Through the analysis of their genomes and comparison with the available genomes of 86 other pigs and wild boars from Europe, Asia, and Africa, totalling 12 populations, we were able to estimate introgression and relatedness and identify unique signatures of selection in this population.

## 2 Materials and methods

### 2.1 DNA extraction and sequencing

Blood samples were collected on FTA cards from six Landim pigs (*n* = 6, 2 female pigs, 4 male pigs) in the Vilankulo locality in the Inhambane province of Mozambique by researchers from the University Eduardo Mondlane. These collections were performed during routine sanitary procedures when farmers brought animals to the station. Samples were collected from animals of different owners. No information regarding pedigree was available. The FTA cards were imported to Portugal after authorisation by Portuguese authorities and in compliance with biosecurity rules.

The DNA was extracted using the phenol–chloroform method (18), and whole-genome sequencing was performed by an external company on an Illumina NovaSeq 6,000 sequencer, generating 150 bp paired-end reads and expected to achieve an average of 10-fold depth.

### 2.2 Read processing and SNP calling

Sequencing adaptors and low-quality reads were pruned using Trimmomatic (19), considering a minimum read length of 50 nt, a maximum read length of 150 nt, and a minimum mean Phred quality score of 20 per read. The resulting high-quality paired reads were mapped to the pig reference genome Sscrofa11.1 (20) using the mem command of the Burrows–Wheeler Aligner (BWA) v.0.7.17 (21). SAMtools v.1.10 (22) was used to sort the output mapped file, and duplicate read pairs were removed using Picard v.2.23.4 “MarkDuplicates”.^1^

After mapping, the BAM output was used to identify variants using “SAMtools and Variant Caller v.1.0.6.” The resulting output was processed with BCFtools v.1.10.2 (23), to remove insertions and deletions (INDELs) and select single-nucleotide polymorphisms (SNPs) located in autosomes and supported by a minimum read depth of 10 and a minimum of 3 reads carrying the alternative allele. Statistics of sequencing data are provided in Supplementary Table S1.

### 2.3 Characterisation of population structure and ancestry

Whole-genome data were obtained from the European Nucleotide Archive (ENA), comprising data from cosmopolitan domestic breeds (Pietrain, Duroc, Landrace, and Large White), the Iberian pig, one Asian domestic breed (Meishan pigs), European and Asian wild boars, and pigs from three African countries—Angola, Nigeria, and Kenya—totalling 86 samples that are referenced in this work as worldwide pigs. Supplementary Table S2 summarises the data used, including one sample of *Sus verrucosus* used as an outgroup.

#### 2.3.1 Principal component analysis

Population structure was evaluated by a principal component analysis, performed using autosomal SNPs. First, SNPs in linkage disequilibrium (LD) were pruned, to reduce redundancy, using PLINK v.1.9073 (24) indep-pairwise option, with a 50 kb window size, steps of 5 SNPs and an *r^2^* threshold of 0.5. The principal components matrix was computed with the pruned SNP data, and principal components with significant eigenvalues were determined with a Tracy–Widom test (25) performed in the R package “AssocTests” v.1.0–1 (26), and visualised with the “Tidyverse” package v.1.3.2 (27).

#### 2.3.2 Phylogenetic and admixture analysis

Phylogenetic analysis was performed to investigate Landim pigs’ ancestry and their relation with worldwide breeds. The synonymous SNPs for all studied samples, plus one *Sus verrucosus* sample as an outgroup, were used to generate a multiple sequence alignment (MSA) using VCF-kit v.0.2.6 (28), which was converted to PHYLIP format using the Python script fasta-to-phylip.py.^2^ Using the PHYLIP v.3.697 software (29), a consensus phylogenetic tree was generated, supported by bootstrap analysis (100 bootstraps), using the Felsenstein distance method (29) and the neighbour-joining option (neighbor), which was plotted with iTOL version 6.8.1 (30).

The ancestry was also evaluated with an admixture analysis. The genotype likelihoods were estimated from the 92 BAM files using ANGSD v.0.93578 (31), based on a SNP *p*-value threshold of 10^−6^. The corresponding genotype likelihood information was then used to estimate admixture proportions using NGSAdmix v.3334 (32), considering a minor allele frequency (MAF) of 0.05. The admixture proportions were estimated for k values between two and twelve (number of populations). The obtained admixture proportions were plotted in R statistical software, package “ggplot2” (33).

#### 2.3.3 Kinship

To assess within-population relatedness, we used PLINK 2.0. For each population, kinship coefficients were estimated using the KING-robust method as implemented in PLINK 2.0 (34).

#### 2.3.4 Patterns of linkage disequilibrium decay

To assess genome-wide patterns of LD in the studied pig populations, we calculated pairwise correlation coefficients (*r^2^*) using PopLDdecay v.3.3 (70). We grouped SNP pairs into bins of 10 kb, with 100 kb intervals and a maximum distance of 250 kb. To visualise the extent and decay of LD, we generated plots illustrating the average *r^2^* values, considering a maximum distance of 250 kb. These plots were created using the R statistical software with the package “ggplot2” (33).

### 2.4 Effect of selection and identification of mutations of interest

#### 2.4.1 Integrated haplotype scores (iHS)

To detect recent selection signatures within populations, the merged VCF file containing 10 domestic pig breeds (LND, ANG, KE, NG, IBN, LW, LR, PI, DU and MS) was first phased with Beagle v.5.4 (35) to identify haplotypes. Next, using the “rehh” R package (36), each population data file was filtered for MAF > 0.05 with the *subset* function. The *scan_hh* function was used to calculate integrated haplotype homozygosity (iHH) values and the iES (integrated extended haplotype homozygosity (EHH) score) for all markers. Finally, we used the *ihh2ihs* function to compute iHS. We used the *calc_candidate_regions* function to identify candidate regions, considering a window size of 10 kb, an overlap of 1 kb, and a minimum of two SNPs below the *p*-value threshold (1×10^−6^).

#### 2.4.2 Cross-population extended haplotype homozygosity (XP-EHH)

To identify regions under selection in distinct populations, we employed the *ies2xpehh* function from the “rehh” package in R (36), with the previously created iES data frame. Specifically, the *p.side = “left”* parameter setting was used, which is tailored to detect regions under selection in the second population that are not observed in the first population. This approach allowed us to pinpoint genomic regions where the Landim pigs displayed EHH and substantial allele frequency differentiation compared to the worldwide breeds. Candidate regions were identified considering a window size of 10 kb, an overlap of 1 kb, and a minimum of five SNPs below the *p*-value threshold (1 × 10^−4^).

#### 2.4.3 Functional impact of SNPs and identified candidate regions

Ensembl’s Variant Effect Predictor (VEP) (37) was used to infer functional effects for identified SNPs. The Genomic Annotation in Livestock for positional candidate *loci* (“GALLO”) R package (38) was used for annotation of genes and quantitative trait *loci* (QTLs) located inside the candidate regions. The Animal QTLdb database (release 53) for pig was used for the analysis (39). Candidate genes were considered to be those overlapping with SNPs or haplotypes. In addition, we checked whether there were any transcription factors or co-transcription factors among the genes located in the candidate regions using the AnimalTFDB 4.0 database (40).

Enrichment analyses for gene ontology (GO) terms were performed with the “gprofiler2” R package (41). Furthermore, the GeneMANIA (v3.5.3) Cytoscape plugin (42, 43) was used to identify other candidate genes that interact with genes located in the confidence regions. For that purpose, orthologous human genes were searched with the “BiomaRt” R package (44). The resulting list of orthologous genes was passed to GeneMANIA to perform a network-based gene set enrichment analysis. Genes without an orthologous pig gene were removed from the network.

## 3 Results

### 3.1 Landim SNP calling and annotation

More than 64 million variants were identified overall in the six Landim samples. After selecting the best quality SNPs and excluding insertions and deletions (INDELs), we identified 9 million autosomal SNPs, with an average frequency of 1 SNP per 0.25 kb, mostly located in intronic regions, followed by the intergenic regions (Table 1). The ratio of transitions and transversions observed was 2.4. Within exonic regions, 38,896 SNPs were annotated as synonymous and 24,283 SNPs were annotated as missense.

**Table 1:** Summary and annotation of autosomal SNPs in Landim pigs.

Category	Value
SNP frequency	1/0.25 kb
Overall total of SNPs	9,003,586
SNPs unique in Landim pigs	842,874
Intron variant	4,318,585
Intergenic variant	3,734,358
Other non-coding SNPs	885,439
Protein coding	65,204
Missense variant	24,283
Stop gained	319
Stop lost	75
Start lost	132
Synonymous variant	40,330
Stop retained variant	38
Coding sequence variant	27

### 3.2 Annotation of missense SNPs

Missense SNPs were present in 9,150 genes, significantly enriched for 421 biological processes (BPs), of which 260 were classified as highly significant based on a false discovery rate (FDR) < 0.01. The summarisation of these 260 enriched BP showed an enrichment of genes related to response to external *stimuli* and their regulation (Supplementary Figure S2). Moreover, the pathway enrichment analysis identified signals involving Rho GTPases as highly significant, which include 142 genes harbouring missense SNPs. Missense SNPs in three genes, *ADGRE3*, *RASSF5*, and in a novel gene *ENSSSCG00000040426*, were classified as having high impact at the translation level.

### 3.3 Characterisation of population structure and ancestry

**Figure 2:**
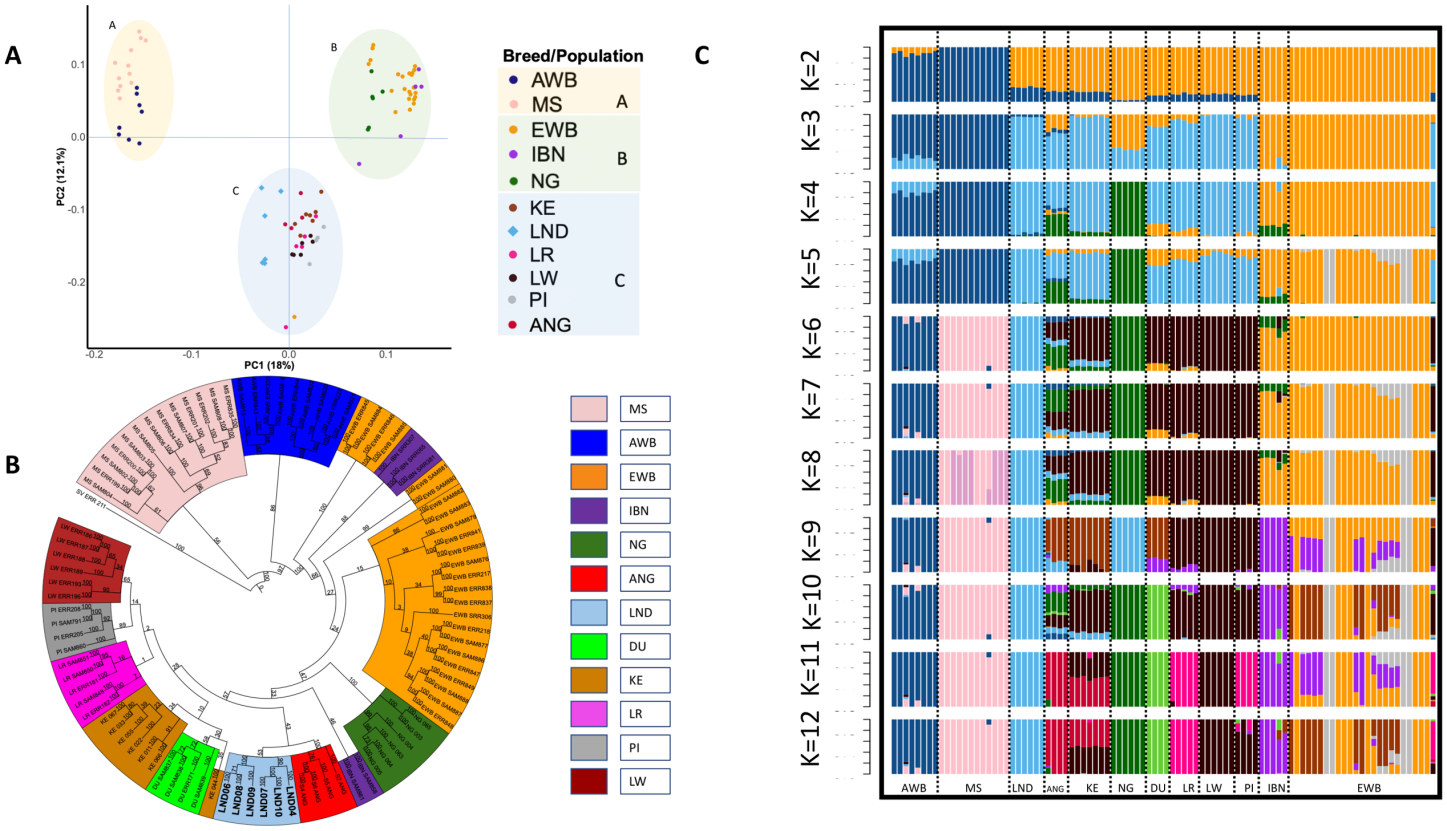
Population structure and ancestry of European, Asian, and African pigs. **(A)** Principal component analysis of 92 samples based on autosomal SNPs, showing the distribution according to principal components 1 and 2. **(B)** Phylogenetic tree of 12 populations, including European, Asian, and African pigs, with *Sus verrucosus* as an outgroup, based on synonymous SNPs. **(C)** Population structure revealed by admixture analysis. EWB, European wild boar; IBN, Iberian; NG, Nigeria; KE, Kenya; LND, Landim; LR, Landrace; LW, Large White; PI, Pietrain; AWB, Asian wild boar; MS, Meishan; DU, Duroc.

Principal component analysis was performed to investigate the population structure in the studied populations. The first (PC1) and second (PC2) principal components explained 30.1% of the total genotype variance. In Figure 2A, PC1 clearly separated European pigs (clusters B and C) from the Asian *Sus scrofa* (cluster A). Among African pigs, Nigerian pigs clustered with European wild boar and Iberian pigs (cluster B), while Landim, Angolan, Kenyan, and the other cosmopolitan pigs grouped together in cluster C.

The phylogenetic tree, rooted using a *Sus verrucosus* sample as an outgroup, allowed us to observe a separation between the European and Asian populations (Figure 2B). Landim pigs were placed within the European clade, closer to Angolan and a subset of Iberian pigs. Nigerian pigs were closer to European wild boar and Iberian samples. Kenyan pigs clustered with Duroc and cosmopolitan European breeds, forming a separate subcluster.

In our admixture analysis (Figure 2C), at *k* = 2, a clear separation between Asian and European pigs was observed. At *k* = 3, three clusters were observed: the first cluster included samples from Asian domestic and wild boars (Asian cluster), the second cluster included African and European domestic pigs, and a third cluster that included Iberian pigs and European wild boars. At *k* = 3, the genomes of Landim and Kenyan pigs shared more than 90% of common ancestry with the genomes of cosmopolitan breeds. At *k* = 6, Landim pigs were differentiated from all other breeds and wild boars, up to *k* = 12. At *k* = 12, Kenyan pigs displayed admixture with Large White or Pietrain and Angolan pigs. Although at *k* = 12 Duroc displayed a different signature, at *k* = 9, it showed joint ancestry with Iberian, Angolan, and Kenyan pigs.

Owing to the unavailability of pedigree information, pairwise kinship coefficients were calculated for individuals within each breed (Supplementary Figure S1). In the case of Landim pigs, values ranged between 0.27 and 0.43. On average, the Landim pigs displayed similar kinship levels to European commercial breeds. These findings are consistent with previously published data and contribute to important studies on genetic diversity and breeding management (Supplementary Table S2).

### 3.4 Effects of selection on Landim pigs

#### 3.4.1 Linkage disequilibrium

**Figure 3:**
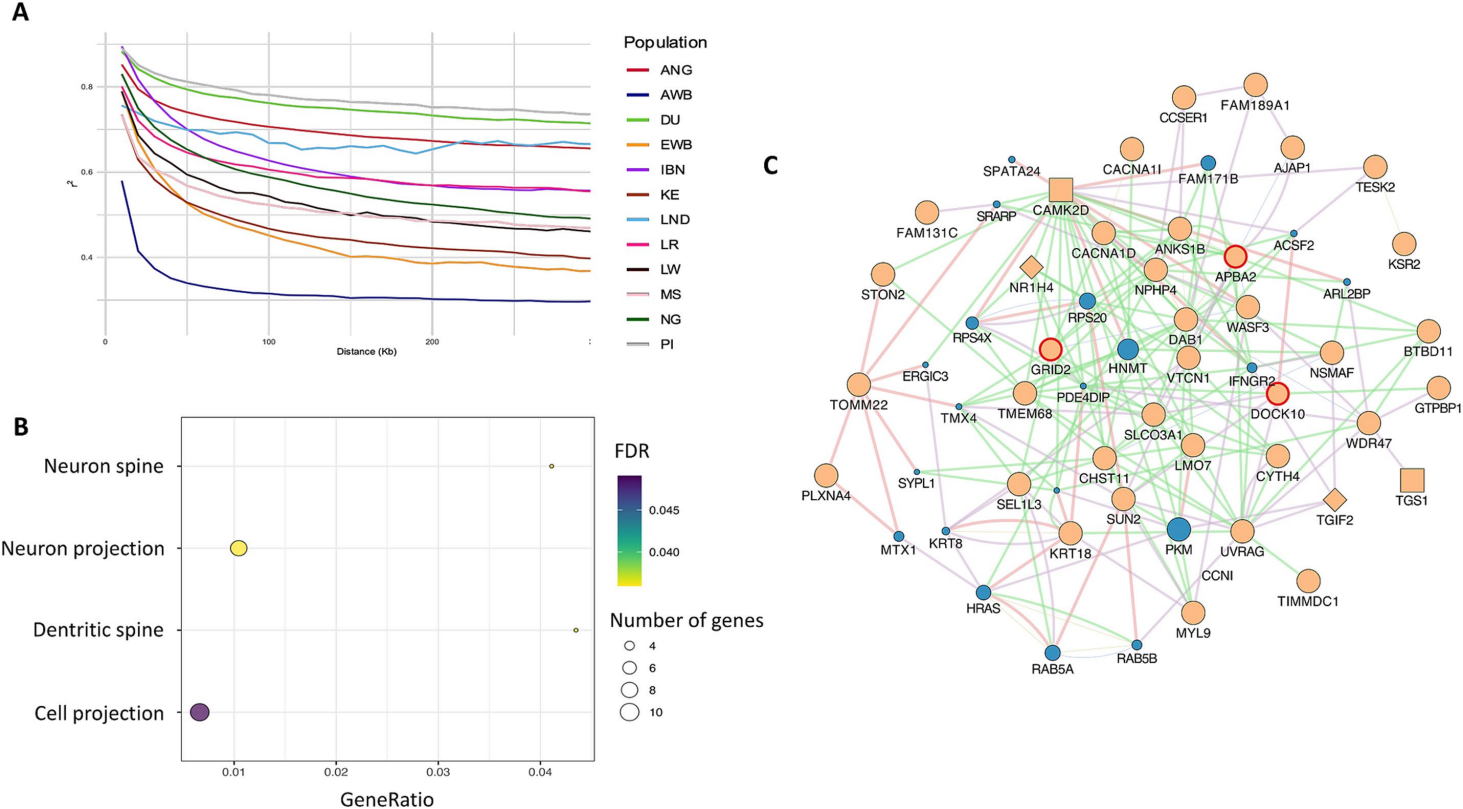
**(A)** Pattern of linkage disequilibrium decay across genomic distances. **(B)** Gene ontology enrichment analysis of iHS candidate genes, showing significant cellular component terms. **(C)** Interaction network of genes identified in iHS regions. Beige = query genes; blue = related genes. Diamond shapes = transcription factors; square shapes = co-transcription factors. Red circles = genes identified in enrichment analysis. Pink lines = physical interactions; Purple lines = co-expression; Blue lines = co-localisation; Green lines = genetic interactions.

Analysing the pattern of linkage disequilibrium (LD) decay across genomic distances provides information on genetic diversity, selection, and population structure. In Figure 3A, it was shown that Landim pigs display high levels of LD at *loci* located at large genomic distances, surpassed only by Duroc and Pietrain, while Kenyan pigs showed the lowest LD among domestic populations, and Asian wild boars exhibited the lowest LD overall.

#### 3.4.2 Integrated haplotype score (iHS)

The integrated haplotype score test is a powerful tool for detecting candidate regions undergoing recent selection, enabling the association of these regions with genes under positive selection. We identified 91 candidate regions spanning 1,639 kb and containing 10,992 SNPs that overlapped with 56 genes (Supplementary Figure S3). Notably, in chromosome SSC6 (*Sus scrofa* chromosome 6), we identified a candidate region with the highest number of SNPs exceeding the threshold (9 SNPs), which overlapped the family with sequence similarity 131, member C (FAM131C) gene. The gene set enrichment analyses identified four cellular component (CC) processes as significant (FDR < 0.05) (Supplementary Table S3). The most significant CC process was neuron projection, related to eight identified genes, whereas cell projection involved 10 genes (Figure 3B). *APBA2*, *GRID2* and *DOCK10* genes were consistently involved in all these processes and displayed a large number of interactions with the other identified genes, as shown in Figure 3C. In this figure, it can be observed that most of the reported interactions are due to epistasis. For *GRID2,* physical interactions were also identified (Figure 3C). As shown in Figure 3C, *GRID2 and APBA2* most likely interact with two transcription factors, *NRIH4* and *TGS1*, and with two co-transcription factors, *CAMK2D* and *TGS1*.

Moreover, these iHS candidate regions overlap with 160 previously identified quantitative trait *loci* (QTLs) (Supplementary Table S4). These QTLs correspond to five categories, of which those for meat and carcass traits and reproduction were the most represented, encompassing 78% and 11%, respectively, and are significantly enriched (*p*-value < 0.001).

#### 3.4.3 Cross-population extended haplotype homozygosity (XP-EHH)

**Figure 4:**
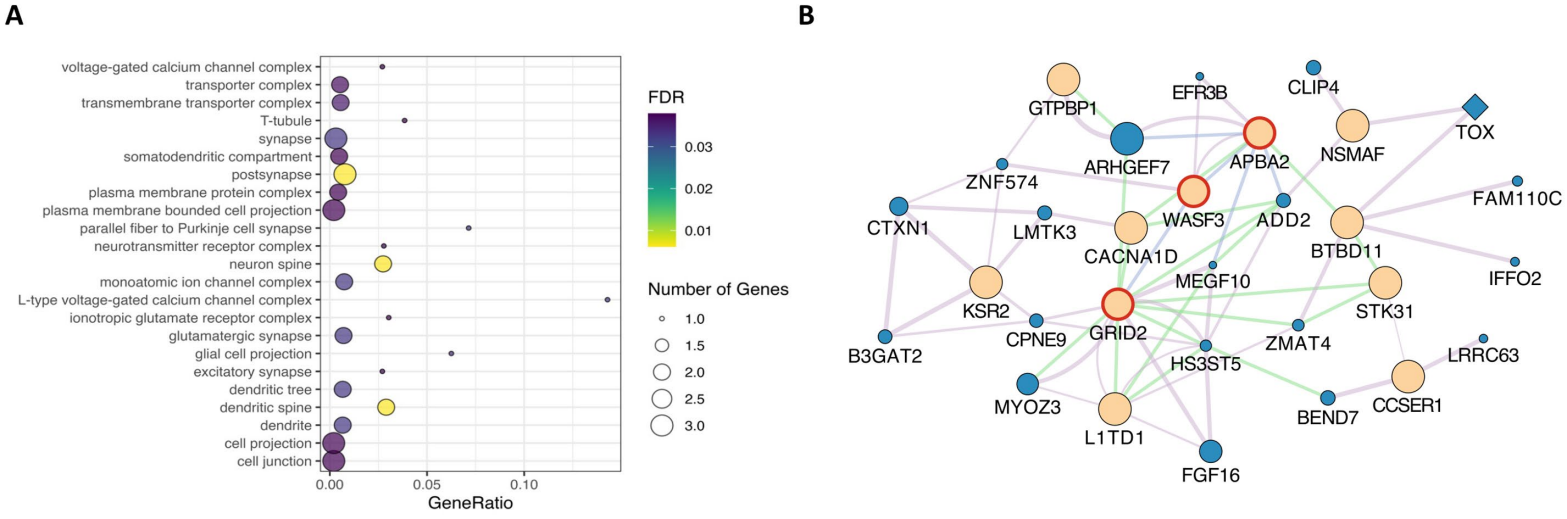
**(A)** Gene ontology enrichment analysis of common genes identified in both iHS and XP-EHH candidate regions, showing significant biological process terms. **(B)** Interaction network of genes identified in iHS regions. Beige = query genes; Blue = related genes. Diamond shapes = transcription factors. Red circle = genes associated in enrichment analysis. Purple lines = co-expression; Blue lines = co-localisation; Green lines = genetic interaction.

We used the XP-EHH method to identify signatures of selection where Landim pigs (LND) displayed extended haplotype homozygosity, while the other nine pig breeds in our study (IBN, LW, LR, PI, DU, ANG, KE, NG, and MS) did not. Table 2 summarises the total number of windows and the cumulative size of these regions, the number of SNPs, and the number of outlier SNPs in these regions. Overall, these regions overlap with 128 genes. The largest number of candidate regions under selection was found when LND pig samples were compared with Iberian pigs (IBN), covering 1,461 kb and containing 1,251 outlier SNPs. By contrast, the fewest candidate regions under selection were found when comparing LND pigs with ANG and MS pigs, with spans of 334 kb and 235 kb, respectively, encompassing seven genes in ANG pigs and five genes in MS pigs. From these, 15 genes (Table 3) were also identified through the iHS analysis (Supplementary Figure S4). The enrichment analysis allowed for the identification of 23 CC processes (Figure 4A). In these CC processes, the genes *APBA2* and *GRID2* were again found and formed an interaction hub, with the prevalence of co-expression interactions along with other genes (Figure 4B).

**Table 2:** Cross-population extended haplotype homozygosity (XP-EHH) results for Landim pigs *versus* the other domestic pig populations.

Breed pair	Number of outlier regions	Regions total extent (kb)	Total SNP count	Outlier SNP count (*p*-value < 1×10^−4^)
LND vs. IBN	67	1,461	9,668	1,251
LND vs. NG	44	1,068	7,431	1,231
LND vs. LR	41	926	7,569	684
LND vs. KE	32	694	4,564	446
LND vs. LW	26	733	4,771	1,070
LND vs. PI	25	492	3,305	294
LND vs. DU	21	444	2,810	485
LND vs. ANG	15	334	2,179	205
LND vs. MS	14	275	1,470	190

**Table 3:** Common genes identified in candidate regions detected by both iHS and XP-EHH methods.

SSC^a^	Ensembl ID	Gene
1	ENSSSCG00000004849	*APBA2*
3	ENSSSCG00000032381	*–*
4	ENSSSCG00000006236	*NSMAF*
5	ENSSSCG00000000095	*GTPBP1*
5	ENSSSCG00000000162	*ABTB3*
8	ENSSSCG00000009197	*GRID2*
8	ENSSSCG00000022986	*CCSER1*
11	ENSSSCG00000009300	*WASF3*
13	ENSSSCG00000011455	*CACNA1D*
13	ENSSSCG00000060753	*IQCF3*
13	ENSSSCG00000055414	*–*
13	ENSSSCG00000043327	*–*
14	ENSSSCG00000009855	*KSR2*
18	ENSSSCG00000016719	*–*
18	ENSSSCG00000016671	*ITPRID1*

a SSC= Sus scrofa chromosome.

Regarding the overlap of XP-EHH candidate regions with known QTLs, the analysis retrieved 250 QTLs; the most abundant categories are meat and carcass (31%) and health (32%), followed by reproduction (15%) and exterior (11%). The cross-analysis to identify QTLs overlapping iHS and XP-EHH candidate regions retrieved nine QTLs (Table 4). These nine QTLs overlap with three of the genes common to both iHS and XP-EHH candidate regions (*NSMAF, GTPBP1, GRID2*).

**Table 4:** Common QTLs found in Landim pigs overlapping iHS and XP-EHH candidate regions and their respective genes.

QTL type	QTL ID	SSC^a^	Start	End	Gene^b^
Meat and Carcass	153,149	4	74,275,00	74,294,00	*NSMAF*
Health	170,961	5	9,260,00	9,279,00	*GTPBP1*
Meat and Carcass	291,259	8	125,880,00	125,904,00	*GRID2*
Meat and Carcass	291,217	8	125,880,00	125,904,00	*GRID2*
Meat and Carcass	291,059	16	29,267,00	29,285,00	–
Meat and Carcass	278,294	16	29,267,00	29,285,00	–
Meat and Carcass	278,295	16	29,267,00	29,285,00	–
Reproduction	257,967	18	48,323,00	48,342,00	ENSSSCG00000016719
Reproduction	257,979	18	48,323,00	48,342,00	ENSSSCG00000016719

a SSC = Sus scrofa chromosome.

b Genes located within QTL boundaries. QTLs with no gene located within QTL boundaries are indicated with a dash (−).

#### 3.4.4 Gene-TF networks

**Figure 5:**
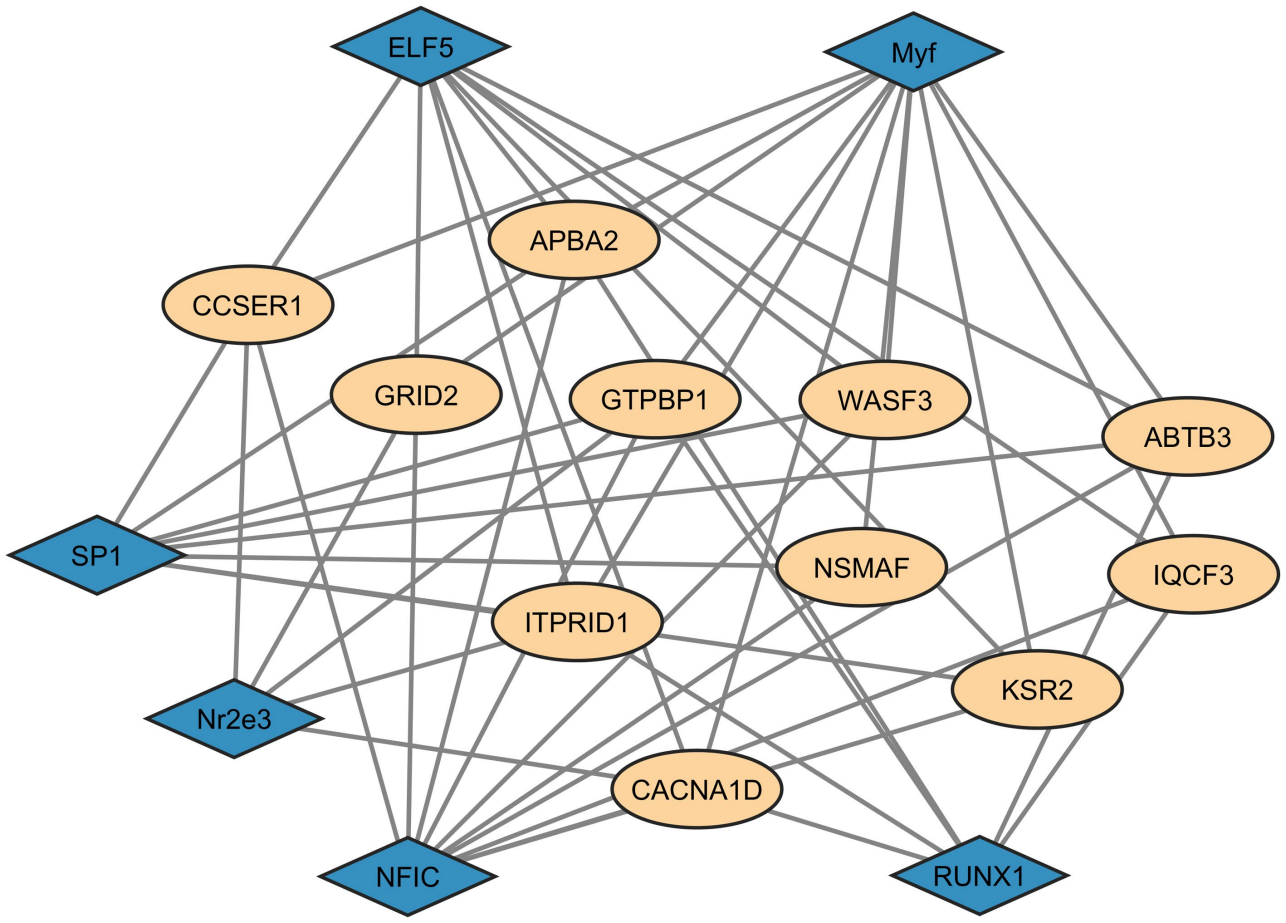
Interaction network of transcription factors for the common genes identified in both iHS and XP-EHH candidate regions. Blue diamonds = transcription factors; Beige circles = common genes in iHS and XP-EHH regions.

Providing evidence of interactions between the identified candidate genes and transcription factor (TF) genes enables the identification of additional *loci* that may influence complex traits. Therefore, for the set of genes detected by both selection methods (iHS and XP-EHH), we conducted an analysis to identify TFs with significant binding affinity (*p* < 0.001). The transcription factors with significant affinity were *ELF5, Myf, RUNX1, SP1, NR2E3*, and *NFIC* (Figure 5). All of these are associated with DNA binding, RNA polymerase activity, and transcriptional regulation. The network analysis revealed multiple interactions between the TFs and the candidate genes.

## 4 Discussion

Although pig breeds in Europe and Asia have been extensively characterised at the genomic level, the same has not yet occurred for African local pigs. These animals can adapt to harsh conditions and possess high levels of genetic diversity. However, little is still known regarding the in-depth characterisation of their genomes. This also applies to Landim pigs from Mozambique, which might have the capacity to survive various disease outbreaks, including African swine fever outbreaks, according to the literature (6).

In this study, we used whole-genome sequencing (WGS) to investigate the relationship of Landim pigs with other pig populations and their level of admixture. Moreover, we identified candidate regions under selection that harbour genes of potential interest for a better understanding of the biological processes underlying adaptive traits. The WGS data generated displayed an average depth of 10x and a mapping rate of at least 99.4%, similar to previous studies in other pig breeds (13, 14, 16) and within the range of the optimal values for SNP calling in pigs (45). The variant calling analysis allowed us to identify more than nine million SNPs in autosomes for the Landim pigs, resulting in an average of 1 SNP per 0.25 kb, crucial for the identification of signatures of selection (46), and showing a ratio of transitions and transversions of 2.4, which is within expected values for the species (47). Despite the high resolution of the whole-genome sequencing data, which provided valuable insights into the genetic makeup of Landim pigs from a single region, our results and interpretations are necessarily based on a small number of individuals. As such, caution should be exercised when attempting to generalise these findings to the wider Landim population, given potential genetic variation across its geographic range. Broader sampling from additional regions is essential to validate and expand upon these observations.

Our results suggest that Landim pigs’ genomes are highly variable, harbouring a higher number of SNPs than their European and Asian counterparts. The functional analyses of missense SNPs revealed a significant enrichment for missense SNPs located in *loci* involved in biological processes related to the response and regulation to external stimuli, which are crucial for survival and reproduction (48). Moreover, missense SNPs with high impact on protein function were identified in genes *ADGRE3* and *RASSF5*. These genes are specifically transcribed in humans in the bone marrow and in lymphoid tissue, with expression enhanced in lymphoid tissue (49). *ADGRE3* is crucial for immune and inflammatory response in humans (50)*. RASSF5* has been identified as a tumour suppressor in several studies focusing on cancers in humans (51). This suggests that Landim pigs harbour unique variants of the species that allow them to adapt their physiology and behaviour to their specific environment.

The results from the phylogenetic analysis splitting Asian and European *Sus scrofa* were in agreement with previous studies regarding pig domestication (52). Regarding African pigs, we observed within the European cluster a sequence of clusters suggesting a higher level of ancestry among European wild boar, Iberian pigs, and Nigerian pigs. Landim pigs are clustered closer to Angolan pigs. Kenyan and Duroc pigs share the same subcluster, and cosmopolitan breeds share another. Our results are consistent with those of Sá et al. (16), who also demonstrated that, in terms of phylogenetic analysis, Angolan pigs share a higher level of ancestry with European pigs, supporting the hypothesis that local African pigs derive from pigs introduced into Africa following domestication in Europe (53). The close relationship between Angolan and Landim pigs is consistent with the historical context of Angola and Mozambique as overseas colonies of Portugal before 1975, and their geographical proximity within the Southern Africa region (54). These factors strongly suggest the possibility of genetic flow between the two populations, highlighting the influence of shared historical and geographical factors on the genetic composition of the Landim pig population. The closeness of Nigerian pigs to Iberian pigs aligns with the historical introduction of Iberian pigs into Nigeria during the 15th century (53). The PCA analysis provided insights into recent gene flow events shaping genetic structure, revealing that Angolan, Landim, and Kenyan pigs cluster more closely with European cosmopolitan breeds. The introduction of such breeds into these regions is well documented, and interbreeding has occurred. Regarding the admixture analysis, from k = 6 onwards, Landim pigs exhibit a distinct genetic signature, consistent with the only previous genetic characterisation of this population using microsatellites (55). In that study, comparisons with European commercial lines, as well as pigs from Namibia and South Africa, similarly highlighted the genetic uniqueness of the Landim. Our admixture results also align with those of Sá et al. (16), who reported a Duroc origin with a notable contribution from African pigs, particularly from Angola, along the West coast.

Owing to logistical constraints in accessing endangered and geographically restricted populations, the number of Landim pig samples available for this study was limited. However, the sample size is comparable to those used in similar whole-genome studies of local or rare pig populations (13, 56). Importantly, the identification of genomic regions under positive selection was conducted using iHS and XP-EHH, which are less sensitive to sample size than other methods and retain an expected accuracy above 90% when supported by high SNP density (approximately 1 SNP per 0.25 kb) (46). Although broader sampling would strengthen population-level inferences, the depth and quality of the current WGS data provide a robust foundation for preliminary insights into local adaptation and selection signatures in Landim pigs.

The iHS method enabled the detection of sites with a rapid increase in the frequency of the derived allele (57). Using this method, we identified 91 regions showing enrichment for gene ontology (GO) terms associated with cellular components (CCs): neuron projection, neuron spine, dendritic spine, and cell projection. Moreover, one of these candidate regions, located on SSC6, displayed a higher density of SNPs and overlapped with genes from the *FAM131C* family. The expression of *FAM131C* genes is enriched in brain tissue, particularly in the cerebellum (49).

The XP-EHH method detects genomic regions under recent positive selection between populations, assuming that selection can be traced by measuring linkage disequilibrium. This method identifies the most overrepresented haplotypes in a population, detecting sites that are entirely or nearly fixed (58). Our analysis identified 128 genes overlapping with XP-EHH candidate regions, including 15 genes overlapping with candidate iHS regions, such as *APBA2* and *GRID2*. The enrichment analysis again highlighted dendritic spine and neuron spine GO terms, associated with CC, which include the genes *APBA2* and *GRID2*. Based on human functional and expression data, *APBA2* is a neuronal adapter protein, and *GRID2* encodes an ionotropic glutamate receptor involved in excitatory neurotransmission, both being predominantly transcribed in the human cerebellum. *APBA2* transcription has also been reported in human immune cells (49). In this final list of candidates, we also found *WASF3*, which is associated with neuronal GO terms, highly expressed in the human brain, and encodes a protein involved in actin cytoskeleton remodelling and cell motility. Similarly, other genes such as *KSR2* and *ITPRID1* show brain-specific transcription in humans (49).

Although these functional inferences are based on human data and may not fully reflect porcine biology, they collectively suggest selective pressure on variants affecting neural circuits. This may be relevant for Landim pigs, which live in semi-feral conditions and rely on foraging and behavioural adaptability—traits potentially governed by neural circuitry. A balanced immune response is also essential for fitness and survival. Recent studies have shown that neural circuits can regulate innate immune responses via neuroimmune pathways (59).

Therefore, while extrapolations from human data require caution, our findings may support the hypothesis that Landim pigs’ genomes have undergone selection at *loci* related to neuroimmune axis function, a system fundamental to both behavioural adaptation and immune response.

While considering the QTL overlapping candidate regions of selection, we find meat, carcass and reproduction QTLs as the most significantly enriched types, thus suggesting that selection has affected genomic regions harbouring genes with a pleiotropic effect. Three of the candidate genes are in these QTL regions. Two are not tissue-specific, whereas *GRID2* is specifically transcribed in the brain and testis of humans (49). As for other genes associated with relevant production traits, *GRID2* is highly pleiotropic, with a total of 81 associations to 39 traits reported so far in humans (49). Transcription of *GRID2* can produce four different transcripts, each of which potentially produces four splice variants (60). The *GRID2* gene is also involved in early developmental processes fundamental to embryogenesis, and in pigs, a recent study has found a significant association with litter size (61).

Regarding the remaining candidate genes, which are specifically expressed in the brain and do not overlap with QTLs, these also present the potential to be highly pleiotropic in pigs. In humans, all these genes have been reported to be associated with numerous traits, with *APBA2* having 17 associations with 10 traits, *WASF3* with 29 associations with 21 traits*, KSR2* with 118 associations with 61 traits, and *ITPRID1* with 20 associations with 14 traits (49).

The identification of TF genes that show a significant relationship with the candidate genes also allowed the identification of other pleiotropic effects. *Elf5* is essential for early embryogenesis and mammary gland development during pregnancy and lactation in humans (62), and it has been associated with litter size and teat number in pigs (61). This could explain the association of the candidate regions with QTLs for reproductive traits. *RUNX1* affects the maturation of porcine oocytes, and *SP1* has an important physiological role during puberty and sexual maturity of pigs (63). *Myf* is associated with the development of fibres and skeletal muscle in pigs (64), which is interestingly related to QTLs for meat and carcass. *NFIC* is active in neurons, glia, and Müller glia–derived progenitor cells in porcine retinas (65).

It has been hypothesised that Landim pigs may display resilience to ASF (6). Although no direct phenotypic or experimental data currently confirm this trait, we explored whether genes located within regions under putative selection have been previously associated with the host response to ASF infection. Interestingly, *KSR2* and *IQCF3* were reported as highly expressed in ASF-infected pigs (66), and *GTPBP1* was found to be significantly upregulated in macrophages during ASF infection (67). In addition, although *CCSER2* and *WASF2* were not identified as direct selection candidates in our analysis, they share interaction partners (*CCSER1* and *WASF3*, respectively) and were reported as upregulated upon ASF infection (67, 68).

Given the pleiotropic nature of the ASF virus and the fact that its key cell entry receptor has not yet been identified (69), these expression patterns may highlight genes of interest in the context of ASF response. However, it is important to stress that these observations are hypothesis-generating. Further functional studies and phenotypic evaluations are required to assess whether genetic variation in these loci contributes to ASF resilience in Landim pigs.

## 5 Conclusion

Our study conducted the first whole-genome sequencing analysis of Landim pigs from Mozambique, revealing high-quality genetic data and a significant number of unique single-nucleotide polymorphisms.

Missense SNP analysis uncovered the potential impact of genetic variations on various biological processes of response and regulation to external *stimuli*, which are crucial for organismal survival and reproduction. An examination of the genetic divergence among the breeds analysed indicates a close genetic relationship between Landim and European pig breeds, likely influenced by historical and geographical factors.

Population structure analysis highlighted the recent influence of genetic inflow from exotic breeds into Landim pigs. Nevertheless, the admixture analysis confirms the uniqueness of the genetic makeup of Landim pigs. The identified signatures of selection highlight key genes that are highly pleiotropic, linked to neuronal circuits, involved in inflammation, and showing significant changes in gene expression upon ASF infection.

These findings improve our understanding of how the Landim pigs might have evolved. They highlight the need for further research focusing on this breed to support the development of breeding and conservation strategies for these local pigs. By revealing what makes these pigs unique and resilient, this study underscores the importance of preserving their genetic heritage and the cultural significance of Landim pigs in the region.

## Data Availability

The datasets presented in this study can be found in online repositories. The names of the repository/repositories and accession number(s) can be found at: https://www.ebi.ac.uk/ena, PRJEB70596, PRJEB49797, PRJEB1683, PRJNA255085, PRJNA691462, PRJEB9922.

## Footnotes

^1^ https://broadinstitute.github.io/picard/

^2^ https://github.com/audy/bioinformatics-hacks
