# Supplementary material for: Genomic characterisation of endangered Landim pigs reveals distinctive features and immune-related selection
Source: Front Vet Sci. 2025 Oct 14;12:1633365. doi: 10.3389/fvets.2025.1633365 (PMC12560241; doi:10.3389/fvets.2025.1633365)
Supplement: Supplementary file 1 [file Data_Sheet_1.docx]

**Supplementary files**

**
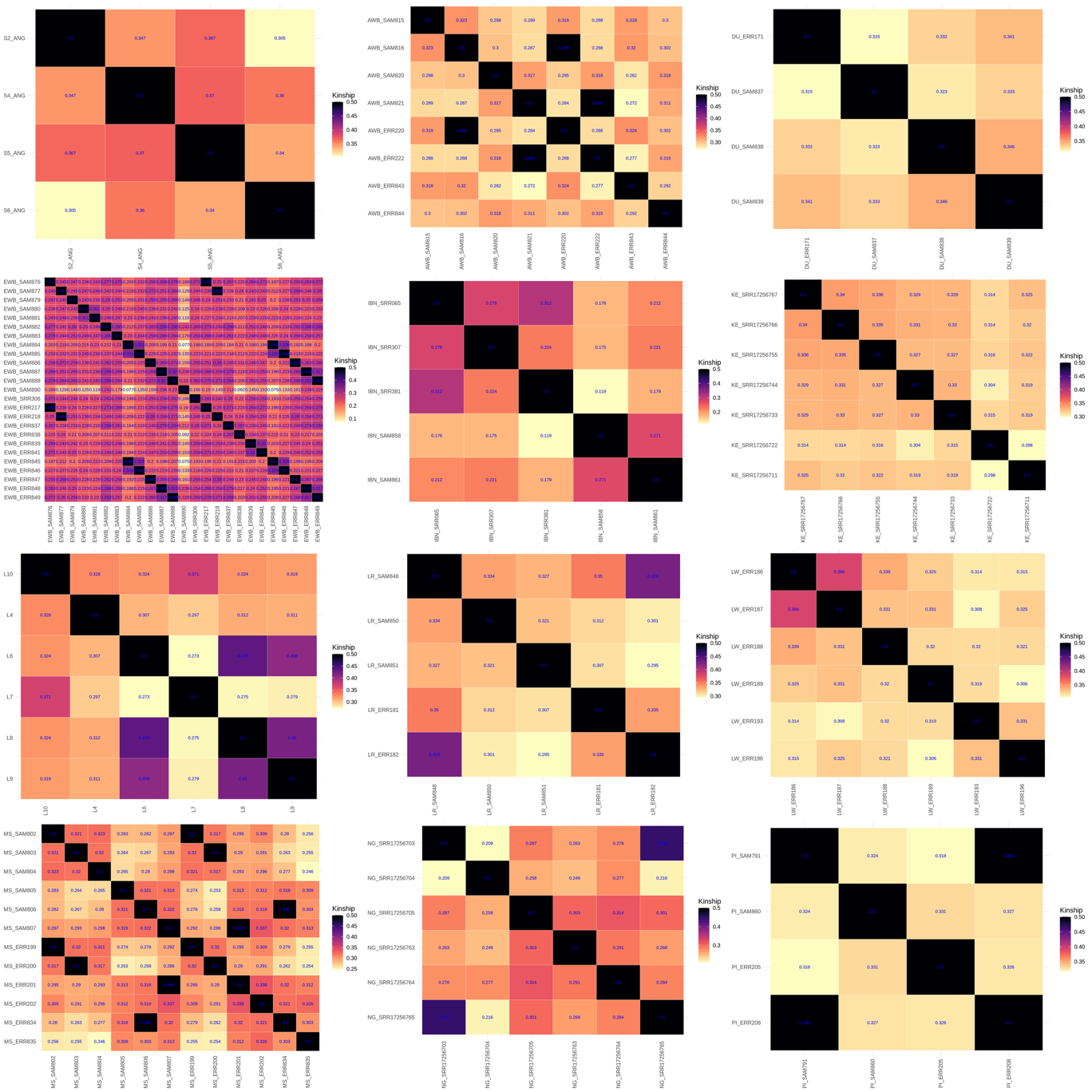
**

**Supplementary Figure S1**. Heatmaps of Genetic Kinship Among Individuals from Diverse Swine Populations". Angola (S2_ANG, S4_ANG, S5_ANG, S6_ANG); Asian Wild Boar - AWB (AWB_SAM815, AWB_SAM816, AWB_SAM820, AWB_SAM821, AWB_ERR220, AWB_ERR222, AWB_ERR843, AWB_ERR844); Duroc (DU_ERR171, DU_SAM837, DU_SAM838, DU_SAM839); European Wild Boar - EWB (EWB_SAM876, EWB_SAM877, EWB_SAM879, EWB_SAM880, EWB_SAM881, EWB_SAM882, EWB_SAM883, EWB_SAM884, EWB_SAM885, EWB_SAM886, EWB_SAM887, EWB_SAM888, EWB_SAM890, EWB_SRR306, EWB_ERR217, EWB_ERR218, EWB_ERR837, EWB_ERR838, EWB_ERR839, EWB_ERR841, EWB_ERR845, EWB_ERR846, EWB_ERR847, EWB_ERR848, EWB_ERR849); Iberian (IBN_SRR065, IBN_SRR307, IBN_SRR381, IBN_SAM858, IBN_SAM861); Kenya (KE_SRR17256767, KE_SRR17256766, KE_SRR17256755, KE_SRR17256744, KE_SRR17256733, KE_SRR17256722, KE_SRR17256711); Landim (L10, L4, L6, L7, L8, L9); Landrace (LR_SAM848, LR_SAM850, LR_SAM851, LR_ERR181, LR_ERR182); Large White (LW_ERR186, LW_ERR187, LW_ERR188, LW_ERR189, LW_ERR193, LW_ERR196); Meishan (MS_SAM802, MS_SAM803, MS_SAM804, MS_SAM805, MS_SAM806, MS_SAM807, MS_ERR199, MS_ERR200, MS_ERR201, MS_ERR202, MS_ERR834, MS_ERR835); Nigeria (NG_SRR17256703, NG_SRR17256704, NG_SRR17256705, NG_SRR17256763, NG_SRR17256764, NG_SRR17256765); Pietrain (PI_SAM791, PI_SAM860, PI_ERR205, PI_ERR208).

**
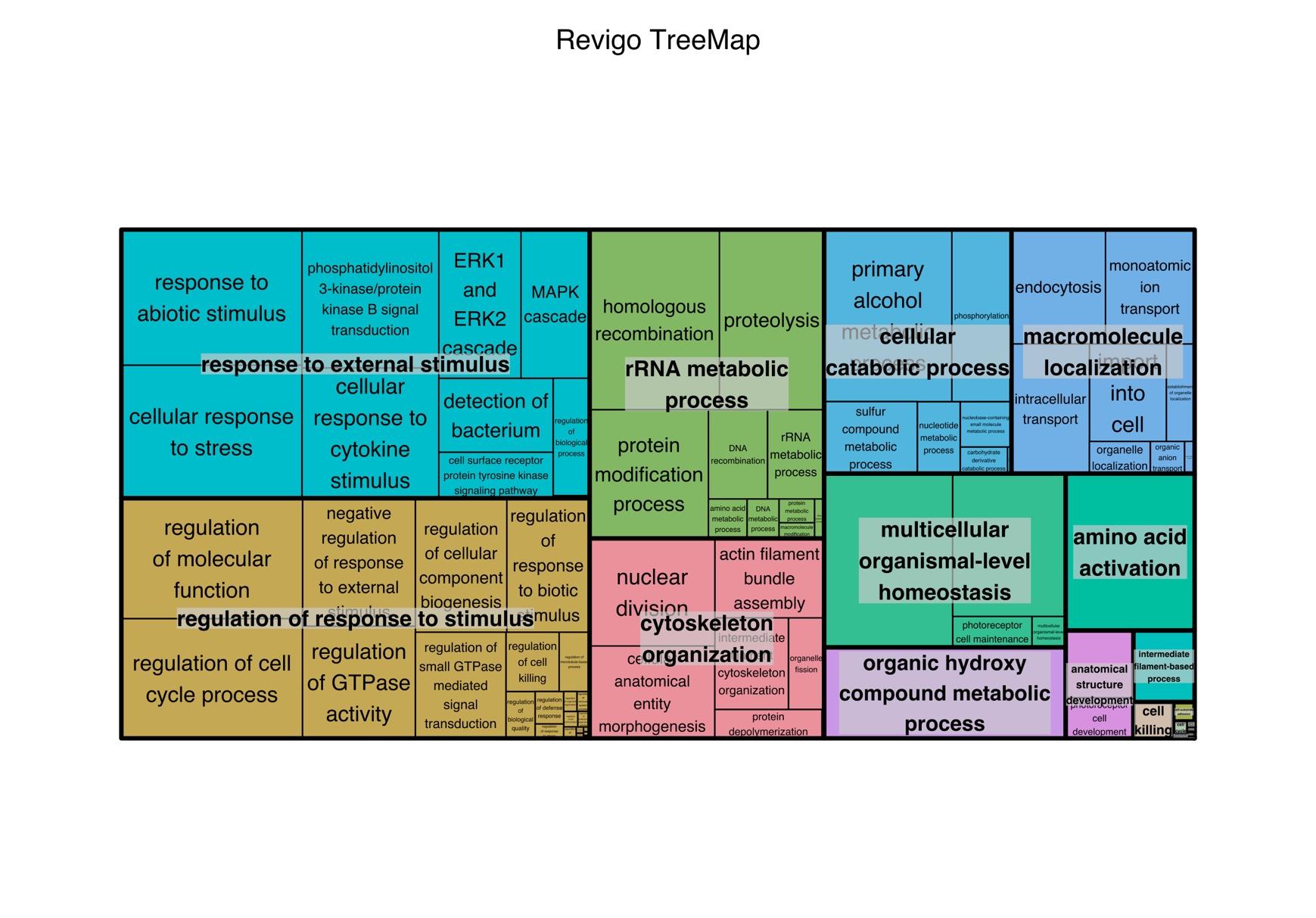
**

Supplementary Figure S2. Summarization of 260 enriched Biological processes associated with Landim missense SNPs.

**
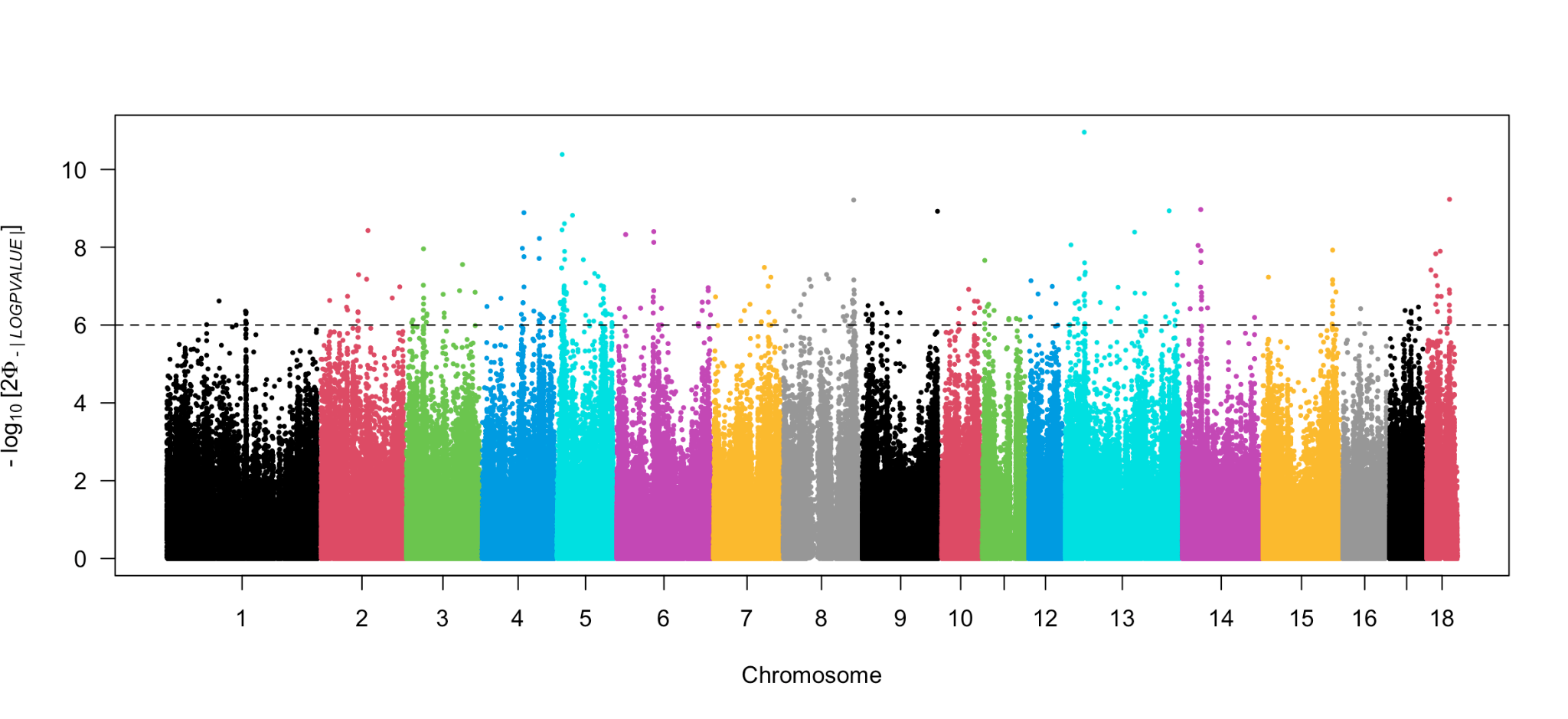
**

Supplementary Figure S3. Manhattan plots of the genome-wide *iHS* analysis.

**
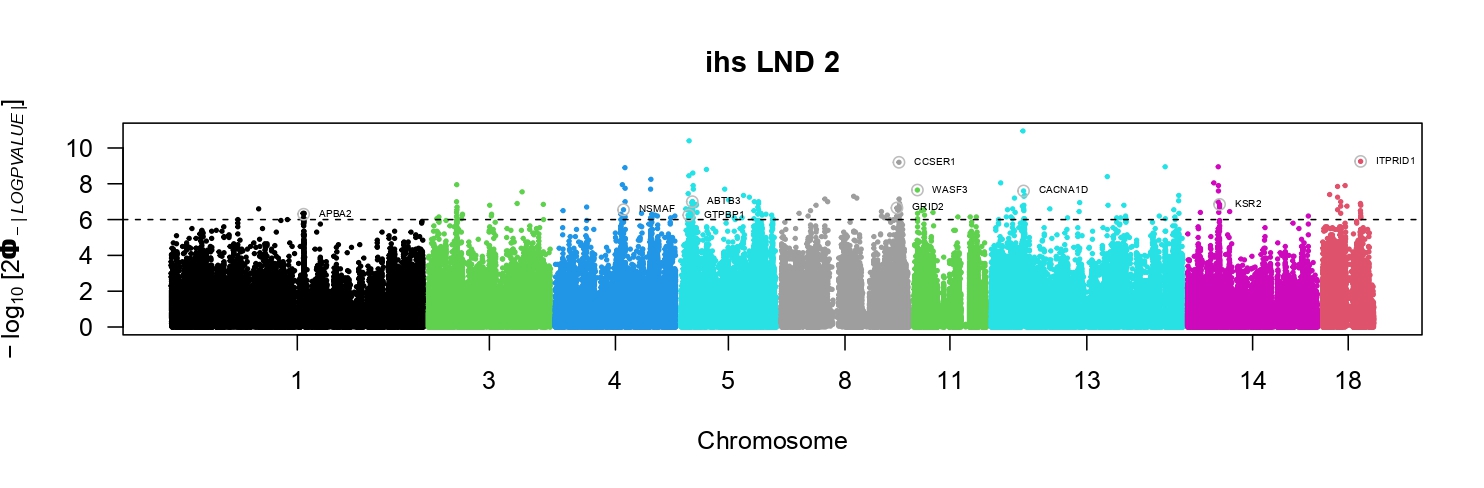
**

Supplementary Figure S4. Manhattan plot of iHS analysis highlighting common genes identified using the XP-EHH method.

##

Supplementary Table S1. Read mapping stats

| **Sample** | **Raw reads** | **Good reads (%)** | **Mapped (%)** | **Covered 1x (%)** | **Covered 10x (%)** | **Depth (x)** |
| --- | --- | --- | --- | --- | --- | --- |
| L4 | 82,916,905 | 99.96 | 99.36 | 97.37 | 22.19 | 7.79 |
| L6 | 108,196,933 | 99.96 | 99.42 | 97.94 | 41.39 | 9.56 |
| L7 | 97,284,090 | 99.96 | 99.46 | 97.41 | 33.06 | 8.82 |
| L8 | 94,534,888 | 99.95 | 99.49 | 97.73 | 30.61 | 8.61 |
| L9 | 61,617,812 | 99.94 | 99.26 | 98 | 48.45 | 10.25 |
|  | 51,415,947 | 99.95 |  |  |  |  |
| L10 | 5,726,808 | 99.95 | 99.53 | 97.68 | 24.59 | 7.97 |
|  | 80,270,150 | 99.95 |  |  |  |  |
| Mean | 72,745,442 | 99.95 | 99.42 | 97.69 | 33.38 | 8.83 |
| SD | 31,757,586 | 0.02 | 0.1 | 0.26 | 10.02 | 0.94 |
| Min | 5,726,808 | 99.9 | 99.26 | 97.37 | 22.19 | 7.79 |
| Max | 108,196,933 | 99.97 | 99.53 | 98 | 48.45 | 10.25 |

Supplementary Table S2. Data source and kinship analysis summary.

| **Breed** | **Acronim** | **Size** | **Study accession** | | **Kinship Min** | **Kinship Max** | **Kinship Mean** | **Kinship SD** |
| --- | --- | --- | --- | --- | --- | --- | --- | --- |
| Angolan | ANG | 4 | PRJEB49797 |  | 0.305 | 0.370 | 0.348 | 0.022 |
| Asian wild boar | AWB | 8 | PRJEB1683 | PRJEB9922 | 0.271 | 0.499 | 0.315 | 0.053 |
| Duroc | DU | 4 | PRJEB1683 | PRJEB9922 | 0.315 | 0.346 | 0.331 | 0.010 |
| European Wild Boar | EWB | 25 | PRJEB1683 | PRJNA255085 | 0.075 | 0.499 | 0.242 | 0.054 |
| Iberian | IBN | 5 | PRJNA255085 | PRJEB9922 | 0.119 | 0.311 | 0.216 | 0.056 |
| Kenian | KE | 7 | PRJNA691462 | | 0.298 | 0.339 | 0.322 | 0.010 |
| Landim (Mozambique) | LND | 6 | PRJEB70596 |  | 0.272 | 0.434 | 0.332 | 0.051 |
| Landrace | LR | 5 | PRJEB1683 | PRJEB9922 | 0.294 | 0.428 | 0.331 | 0.037 |
| Largewhite | LW | 6 | PRJEB1683 |  | 0.305 | 0.386 | 0.326 | 0.018 |
| Meishan | MS | 12 | PRJEB1683 | PRJEB9922 | 0.246 | 0.499 | 0.308 | 0.054 |
| Nigerian | NG | 6 | PRJNA691462 | | 0.209 | 0.446 | 0.282 | 0.052 |
| Pietrain | PI | 4 | PRJEB1683 | PRJEB9922 | 0.317 | 0.498 | 0.354 | 0.067 |
| Sus Verrucosus | SVV | 1 | PRJEB1683 |  | NA | NA | NA | NA |
| Total |  | 93 |  |  |  |  |  |  |

Supplementary Table S3. Gene set enrichment analyses of genes identified in iHS candidate regions

| **N** | **GO.ID** | **Description** | **Term Size** | **p.Val** | **GENES** | **Number of Genes** | **FDR** |
| --- | --- | --- | --- | --- | --- | --- | --- |
| 1 | GO:0043005 | Neuron projection | 765 | 0.03593923 | APBA2 | 8 | 0.03593923 |
|  |  |  |  |  | WDR47 |  |  |
|  |  |  |  |  | CACNA1I |  |  |
|  |  |  |  |  | NPHP4 |  |  |
|  |  |  |  |  | STON2 |  |  |
|  |  |  |  |  | CAMK2D |  |  |
|  |  |  |  |  | GRID2 |  |  |
|  |  |  |  |  | DOCK10 |  |  |
| 2 | GO:0043197 | Dendritic spine | 69 | 0.03593923 | APBA2 | 3 | 0.03593923 |
|  |  |  |  |  | GRID2 |  |  |
|  |  |  |  |  | DOCK10 |  |  |
| 3 | GO:0044309 | Neuron spine | 73 | 0.03593923 | APBA2 | 3 | 0.03593923 |
|  |  |  |  |  | GRID2 |  |  |
|  |  |  |  |  | DOCK10 |  |  |
| 4 | GO:0042995 | Cell projection | 1506 | 0.04907691 | APBA2 | 10 | 0.04907691 |
|  |  |  |  |  | WDR47 |  |  |
|  |  |  |  |  | CACNA1I |  |  |
|  |  |  |  |  | ENSSSCG00000022925 |  |  |
|  |  |  |  |  | NPHP4 |  |  |
|  |  |  |  |  | STON2 |  |  |
|  |  |  |  |  | CAMK2D |  |  |
|  |  |  |  |  | GRID2 |  |  |
|  |  |  |  |  | WASF3 |  |  |
|  |  |  |  |  | DOCK10 |  |  |

Supplementary Table S4. QTL identified in iHS candidate regions

| **CHR** | **QTL type** | **Start pos** | **End pos** | **QTL ID** | **Name** | **BaseTrait** | **P-value** |
| --- | --- | --- | --- | --- | --- | --- | --- |
| 2 | Meat and Carcass | 133184384 | 133184388 | 292666 | Longissimus muscle depth | Longissimus muscle depth | <0.05 |
| 2 | Reproduction | 53052155 | 53052159 | 293644 | Offspring number | Offspring number | <0.05 |
| 3 | Health | 31484732 | 31484736 | 129271 | CD8-negative leukocyte percentage | CD8-negative leukocyte percentage | <1 |
| 3 | Health | 31484732 | 31484736 | 129755 | CD8-positive leukocyte percentage | CD8-positive leukocyte percentage | <1 |
| 4 | Meat and Carcass | 73283395 | 73283399 | 153065 | Head weight | Head weight | <0.05 |
| 4 | Meat and Carcass | 74273496 | 74273500 | 153149 | Head weight | Head weight | <0.05 |
| 4 | Meat and Carcass | 73283395 | 73283399 | 153169 | Tongue weight | Tongue weight | <0.05 |
| 4 | Meat and Carcass | 75479604 | 75479608 | 153554 | Subcutaneous fat thickness | Subcutaneous fat thickness | <0.05 |
| 4 | Meat and Carcass | 75486436 | 75486440 | 160026 | Number of ribs | Number of ribs | <0.05 |
| 4 | Meat and Carcass | 73283395 | 73283399 | 168342 | Cooking loss | Cooking loss | 4,16E-05 |
| 4 | Production | 75479604 | 75479608 | 173205 | Body circumference | Body circumference | <0.05 |
| 4 | Meat and Carcass | 75479604 | 75479608 | 173209 | Subcutaneous fat thickness | Subcutaneous fat thickness | <0.05 |
| 4 | Meat and Carcass | 75479121 | 75479125 | 288250 | Subcutaneous fat thickness | Subcutaneous fat thickness | <0.05 |
| 4 | Meat and Carcass | 75486510 | 75486514 | 288251 | Subcutaneous fat thickness | Subcutaneous fat thickness | <0.05 |
| 4 | Meat and Carcass | 75486538 | 75486542 | 288252 | Subcutaneous fat thickness | Subcutaneous fat thickness | <0.05 |
| 4 | Meat and Carcass | 75486837 | 75486841 | 288253 | Subcutaneous fat thickness | Subcutaneous fat thickness | <0.05 |
| 4 | Meat and Carcass | 75486897 | 75486901 | 288254 | Subcutaneous fat thickness | Subcutaneous fat thickness | <0.05 |
| 4 | Meat and Carcass | 75460655 | 75460659 | 288307 | Subcutaneous fat thickness | Subcutaneous fat thickness | 2,33E-07 |
| 4 | Meat and Carcass | 75460657 | 75460661 | 288308 | Subcutaneous fat thickness | Subcutaneous fat thickness | 2,33E-07 |
| 4 | Meat and Carcass | 75472661 | 75472665 | 291294 | Subcutaneous fat thickness | Subcutaneous fat thickness | <0.05 |
| 4 | Reproduction | 103178453 | 103178457 | 292184 | Litter weight, piglets born alive | Litter weight, piglets born alive | <0.05 |
| 4 | Reproduction | 103171887 | 103171891 | 294691 | Teat number | Teat number | <0.05 |
| 5 | Exterior | 4131294 | 4131298 | 21973 | Gait score (front) | Gait score (front) | 1,50E-05 |
| 5 | Meat and Carcass | 10448734 | 10448738 | 23365 | Subcutaneous fat thickness | Subcutaneous fat thickness | 3,61E-05 |
| 5 | Production | 10448734 | 10448738 | 28830 | Average daily gain | Average daily gain | 1,10E-03 |
| 5 | Reproduction | 4131294 | 4131298 | 64739 | Litter size | Litter size | NA |
| 5 | Exterior | 8681383 | 8681387 | 124367 | Coping behavior | Coping behavior | 0,0464 |
| 5 | Exterior | 8681383 | 8681387 | 124676 | Coping behavior | Coping behavior | 0,0177 |
| 5 | Production | 89480428 | 89480432 | 170636 | Average daily gain | Average daily gain | NA |
| 5 | Production | 89480428 | 89480432 | 170641 | Days to 90 kg | Days to 90 kg | NA |
| 5 | Health | 9259249 | 9259253 | 170961 | Melanoma susceptibility | Melanoma susceptibility | <5E-5 |
| 5 | Reproduction | 13940473 | 13940477 | 287075 | Teat number | Teat number | 1,23E-05 |
| 5 | Meat and Carcass | 84949178 | 84949182 | 290560 | Subcutaneous fat thickness | Subcutaneous fat thickness | <0.05 |
| 5 | Reproduction | 8652085 | 8652089 | 291903 | Offspring number | Offspring number | <0.05 |
| 5 | Reproduction | 63058258 | 63058262 | 292270 | Litter weight, piglets born alive | Litter weight, piglets born alive | <0.05 |
| 5 | Reproduction | 8656151 | 8656155 | 293869 | Sperm count | Sperm count | <0.05 |
| 6 | Exterior | 154970952 | 154970956 | 64729 | Thoracic vertebra number | Thoracic vertebra number | NA |
| 6 | Meat and Carcass | 65527500 | 65527504 | 160984 | Muscle conductivity | Muscle conductivity | 6,31E-06 |
| 6 | Meat and Carcass | 75195770 | 75195774 | 161055 | Muscle conductivity | Muscle conductivity | 1,44E-05 |
| 6 | Production | 75202123 | 75202127 | 194805 | Bone mineral density | Bone mineral density | 1,05E-06 |
| 6 | Meat and Carcass | 63612726 | 66429264 | 238365 | Lean meat percentage | Lean meat percentage | NA |
| 6 | Meat and Carcass | 165918309 | 165918313 | 262279 | Longissimus muscle area | Longissimus muscle area | 3,93E-07 |
| 6 | Meat and Carcass | 165924142 | 165924146 | 262296 | Longissimus muscle area | Longissimus muscle area | 7,65E-08 |
| 6 | Meat and Carcass | 165924108 | 165924112 | 262380 | Longissimus muscle area | Longissimus muscle area | 7,65E-08 |
| 6 | Meat and Carcass | 165927018 | 165927022 | 262382 | Longissimus muscle area | Longissimus muscle area | 7,83E-08 |
| 6 | Reproduction | 165939061 | 165939065 | 295621 | Weaning to estrus interval | Weaning to estrus interval | <0.05 |
| 7 | Meat and Carcass | 92418232 | 92418236 | 268055 | Number of ribs | Number of ribs | <0.05 |
| 7 | Reproduction | 86810079 | 86810083 | 295174 | Teat number | Teat number | <0.05 |
| 8 | Reproduction | 53332016 | 53332020 | 95547 | Teat number | Teat number | <0.05 |
| 8 | Meat and Carcass | 108943742 | 108943746 | 139256 | Subcutaneous fat thickness | Subcutaneous fat thickness | <0.05 |
| 8 | Meat and Carcass | 70533867 | 70533871 | 161256 | Muscle conductivity | Muscle conductivity | <0.05 |
| 8 | Meat and Carcass | 125907460 | 125907464 | 291217 | Subcutaneous fat thickness | Subcutaneous fat thickness | <0.05 |
| 8 | Meat and Carcass | 125906881 | 125906885 | 291259 | Subcutaneous fat thickness | Subcutaneous fat thickness | <0.05 |
| 8 | Meat and Carcass | 19521265 | 19521269 | 291740 | Lean cuts percentage | Lean cuts percentage | <0.05 |
| 8 | Reproduction | 23135293 | 23135297 | 294505 | Teat number | Teat number | <0.05 |
| 8 | Reproduction | 23135293 | 23135297 | 294506 | Teat number | Teat number | <0.05 |
| 9 | Meat and Carcass | 119438703 | 119438707 | 23301 | Subcutaneous fat thickness | Subcutaneous fat thickness | 5,26E-06 |
| 9 | Reproduction | 137095795 | 137095799 | 130319 | Litter size | Litter size | NA |
| 9 | Meat and Carcass | 10311853 | 10311857 | 169958 | Myristic acid content | Myristic acid content | NA |
| 9 | Meat and Carcass | 10311853 | 10311857 | 169959 | Myristic acid content | Myristic acid content | NA |
| 9 | Meat and Carcass | 10331865 | 10331869 | 169960 | Myristic acid content | Myristic acid content | NA |
| 9 | Meat and Carcass | 10331865 | 10331869 | 169961 | Myristic acid content | Myristic acid content | NA |
| 10 | Exterior | 67283548 | 67283552 | 124436 | Coping behavior | Coping behavior | 0,0378 |
| 10 | Exterior | 67283548 | 67283552 | 124759 | Coping behavior | Coping behavior | 0,0337 |
| 10 | Exterior | 67283548 | 67283552 | 124956 | Coping behavior | Coping behavior | 0,0064 |
| 10 | Exterior | 67283548 | 67283552 | 124957 | Coping behavior | Coping behavior | 0,0044 |
| 10 | Reproduction | 67269928 | 67269932 | 295200 | Teat number | Teat number | <0.05 |
| 16 | Exterior | 29292555 | 29292559 | 64666 | Lumbar vertebra number | Lumbar vertebra number | NA |
| 16 | Meat and Carcass | 29292555 | 29292559 | 101669 | Arachidic acid to stearic acid ratio | Arachidic acid to stearic acid ratio | 5,01E-08 |
| 16 | Meat and Carcass | 29283339 | 29283343 | 278294 | Meat color | Meat color | <0.05 |
| 16 | Meat and Carcass | 29283979 | 29283983 | 278295 | Meat color | Meat color | <0.05 |
| 16 | Meat and Carcass | 29284379 | 29284383 | 278296 | Meat color | Meat color | <0.05 |
| 16 | Meat and Carcass | 29284485 | 29284489 | 278297 | Meat color | Meat color | <0.05 |
| 16 | Meat and Carcass | 29284630 | 29284634 | 278298 | Meat color | Meat color | <0.05 |
| 16 | Meat and Carcass | 29284745 | 29284749 | 278299 | Meat color | Meat color | <0.05 |
| 16 | Meat and Carcass | 29284794 | 29284798 | 278300 | Meat color | Meat color | <0.05 |
| 16 | Meat and Carcass | 29284801 | 29284805 | 278301 | Meat color | Meat color | <0.05 |
| 16 | Meat and Carcass | 29284807 | 29284811 | 278302 | Meat color | Meat color | <0.05 |
| 16 | Meat and Carcass | 29284896 | 29284900 | 278303 | Meat color | Meat color | <0.05 |
| 16 | Meat and Carcass | 29284992 | 29284996 | 278304 | Meat color | Meat color | <0.05 |
| 16 | Meat and Carcass | 29285298 | 29285302 | 278305 | Meat color | Meat color | <0.05 |
| 16 | Meat and Carcass | 29285529 | 29285533 | 278306 | Meat color | Meat color | <0.05 |
| 16 | Meat and Carcass | 29285569 | 29285573 | 278307 | Meat color | Meat color | <0.05 |
| 16 | Meat and Carcass | 29285585 | 29285589 | 278308 | Meat color | Meat color | <0.05 |
| 16 | Meat and Carcass | 29285641 | 29285645 | 278309 | Meat color | Meat color | <0.05 |
| 16 | Meat and Carcass | 29285662 | 29285666 | 278310 | Meat color | Meat color | <0.05 |
| 16 | Meat and Carcass | 29285700 | 29285704 | 278311 | Meat color | Meat color | <0.05 |
| 16 | Meat and Carcass | 29285929 | 29285933 | 278312 | Meat color | Meat color | <0.05 |
| 16 | Meat and Carcass | 29286022 | 29286026 | 278313 | Meat color | Meat color | <0.05 |
| 16 | Meat and Carcass | 29286098 | 29286102 | 278314 | Meat color | Meat color | <0.05 |
| 16 | Meat and Carcass | 29286106 | 29286110 | 278315 | Meat color | Meat color | <0.05 |
| 16 | Meat and Carcass | 29286135 | 29286139 | 278316 | Meat color | Meat color | <0.05 |
| 16 | Meat and Carcass | 29286147 | 29286151 | 278317 | Meat color | Meat color | <0.05 |
| 16 | Meat and Carcass | 29286217 | 29286221 | 278318 | Meat color | Meat color | <0.05 |
| 16 | Meat and Carcass | 29286400 | 29286404 | 278319 | Meat color | Meat color | <0.05 |
| 16 | Meat and Carcass | 29286455 | 29286459 | 278320 | Meat color | Meat color | <0.05 |
| 16 | Meat and Carcass | 29286551 | 29286555 | 278321 | Meat color | Meat color | <0.05 |
| 16 | Meat and Carcass | 29286868 | 29286872 | 278322 | Meat color | Meat color | <0.05 |
| 16 | Meat and Carcass | 29286896 | 29286900 | 278323 | Meat color | Meat color | <0.05 |
| 16 | Meat and Carcass | 29286910 | 29286914 | 278324 | Meat color | Meat color | <0.05 |
| 16 | Meat and Carcass | 29287272 | 29287276 | 278325 | Meat color | Meat color | <0.05 |
| 16 | Meat and Carcass | 29287302 | 29287306 | 278326 | Meat color | Meat color | <0.05 |
| 16 | Meat and Carcass | 29287357 | 29287361 | 278327 | Meat color | Meat color | <0.05 |
| 16 | Meat and Carcass | 29287533 | 29287537 | 278328 | Meat color | Meat color | <0.05 |
| 16 | Meat and Carcass | 29287881 | 29287885 | 278329 | Meat color | Meat color | <0.05 |
| 16 | Meat and Carcass | 29287952 | 29287956 | 278330 | Meat color | Meat color | <0.05 |
| 16 | Meat and Carcass | 29287980 | 29287984 | 278331 | Meat color | Meat color | <0.05 |
| 16 | Meat and Carcass | 29288249 | 29288253 | 278332 | Meat color | Meat color | <0.05 |
| 16 | Meat and Carcass | 29288357 | 29288361 | 278333 | Meat color | Meat color | <0.05 |
| 16 | Meat and Carcass | 29288358 | 29288362 | 278334 | Meat color | Meat color | <0.05 |
| 16 | Meat and Carcass | 29288548 | 29288552 | 278335 | Meat color | Meat color | <0.05 |
| 16 | Meat and Carcass | 29288582 | 29288586 | 278336 | Meat color | Meat color | <0.05 |
| 16 | Meat and Carcass | 29288653 | 29288657 | 278337 | Meat color | Meat color | <0.05 |
| 16 | Meat and Carcass | 29288840 | 29288844 | 278338 | Meat color | Meat color | <0.05 |
| 16 | Meat and Carcass | 29288977 | 29288981 | 278339 | Meat color | Meat color | <0.05 |
| 16 | Meat and Carcass | 29288980 | 29288984 | 278340 | Meat color | Meat color | <0.05 |
| 16 | Meat and Carcass | 29289225 | 29289229 | 278341 | Meat color | Meat color | <0.05 |
| 16 | Meat and Carcass | 29289345 | 29289349 | 278342 | Meat color | Meat color | <0.05 |
| 16 | Meat and Carcass | 29289603 | 29289607 | 278343 | Meat color | Meat color | <0.05 |
| 16 | Meat and Carcass | 29289981 | 29289985 | 278344 | Meat color | Meat color | <0.05 |
| 16 | Meat and Carcass | 29290100 | 29290104 | 278345 | Meat color | Meat color | <0.05 |
| 16 | Meat and Carcass | 29290848 | 29290852 | 278346 | Meat color | Meat color | <0.05 |
| 16 | Meat and Carcass | 29291174 | 29291178 | 278347 | Meat color | Meat color | <0.05 |
| 16 | Meat and Carcass | 29291355 | 29291359 | 278348 | Meat color | Meat color | <0.05 |
| 16 | Meat and Carcass | 29291563 | 29291567 | 278349 | Meat color | Meat color | <0.05 |
| 16 | Meat and Carcass | 29291685 | 29291689 | 278350 | Meat color | Meat color | <0.05 |
| 16 | Meat and Carcass | 29292356 | 29292360 | 278351 | Meat color | Meat color | <0.05 |
| 16 | Meat and Carcass | 29292385 | 29292389 | 278352 | Meat color | Meat color | <0.05 |
| 16 | Meat and Carcass | 29292458 | 29292462 | 278353 | Meat color | Meat color | <0.05 |
| 16 | Meat and Carcass | 29292555 | 29292559 | 278354 | Meat color | Meat color | <0.05 |
| 16 | Meat and Carcass | 29292840 | 29292844 | 278355 | Meat color | Meat color | <0.05 |
| 16 | Meat and Carcass | 29292842 | 29292846 | 278356 | Meat color | Meat color | <0.05 |
| 16 | Meat and Carcass | 29292862 | 29292866 | 278357 | Meat color | Meat color | <0.05 |
| 16 | Meat and Carcass | 29292864 | 29292868 | 278358 | Meat color | Meat color | <0.05 |
| 16 | Meat and Carcass | 29292867 | 29292871 | 278359 | Meat color | Meat color | <0.05 |
| 16 | Meat and Carcass | 29292886 | 29292890 | 278360 | Meat color | Meat color | <0.05 |
| 16 | Meat and Carcass | 29293082 | 29293086 | 278361 | Meat color | Meat color | <0.05 |
| 16 | Meat and Carcass | 29293314 | 29293318 | 278362 | Meat color | Meat color | <0.05 |
| 16 | Meat and Carcass | 29293326 | 29293330 | 278363 | Meat color | Meat color | <0.05 |
| 16 | Meat and Carcass | 29293361 | 29293365 | 278364 | Meat color | Meat color | <0.05 |
| 16 | Meat and Carcass | 29293377 | 29293381 | 278365 | Meat color | Meat color | <0.05 |
| 16 | Meat and Carcass | 29293557 | 29293561 | 278366 | Meat color | Meat color | <0.05 |
| 16 | Meat and Carcass | 29293599 | 29293603 | 278367 | Meat color | Meat color | <0.05 |
| 16 | Meat and Carcass | 29293713 | 29293717 | 278368 | Meat color | Meat color | <0.05 |
| 16 | Meat and Carcass | 29293729 | 29293733 | 278369 | Meat color | Meat color | <0.05 |
| 16 | Meat and Carcass | 29293789 | 29293793 | 278370 | Meat color | Meat color | <0.05 |
| 16 | Meat and Carcass | 29293944 | 29293948 | 278371 | Meat color | Meat color | <0.05 |
| 16 | Meat and Carcass | 29294132 | 29294136 | 278372 | Meat color | Meat color | <0.05 |
| 16 | Meat and Carcass | 29294417 | 29294421 | 278373 | Meat color | Meat color | <0.05 |
| 16 | Meat and Carcass | 29294460 | 29294464 | 278374 | Meat color | Meat color | <0.05 |
| 16 | Meat and Carcass | 29294661 | 29294665 | 278375 | Meat color | Meat color | <0.05 |
| 16 | Meat and Carcass | 29294794 | 29294798 | 278376 | Meat color | Meat color | <0.05 |
| 16 | Meat and Carcass | 29294814 | 29294818 | 278377 | Meat color | Meat color | <0.05 |
| 16 | Meat and Carcass | 29294911 | 29294915 | 278378 | Meat color | Meat color | <0.05 |
| 16 | Meat and Carcass | 29294989 | 29294993 | 278379 | Meat color | Meat color | <0.05 |
| 16 | Meat and Carcass | 29279985 | 29279989 | 291059 | Subcutaneous fat thickness | Subcutaneous fat thickness | <0.05 |
| 16 | Reproduction | 59546459 | 59546463 | 294349 | Teat number | Teat number | <0.05 |
| 18 | Health | 41575078 | 41575082 | 127152 | Red blood cell count | Red blood cell count | <1 |
| 18 | Reproduction | 48316682 | 48316686 | 257967 | Teat number | Teat number | <0.05 |
| 18 | Reproduction | 48316682 | 48316686 | 257979 | Teat number | Teat number | <0.05 |
